# Genetic identification and diversity of stocks of the African bonytongue, *Heterotis niloticus* (Osteoglossiformes: Arapaiminae), in Nigeria, West Africa

**DOI:** 10.1038/s41598-022-12428-6

**Published:** 2022-05-19

**Authors:** Tofunmi E. Oladimeji, Isabel C. Caballero, Mariana Mateos, Michael O. Awodiran, Kirk O. Winemiller, Alphonse Adite, Luis A. Hurtado

**Affiliations:** 1 Department of Zoology, Obafemi Awolowo University, Ile-Ife, Nigeria; 2 Department of Ecology and Conservation Biology, Texas A&M University, College Station, TX USA; 3 Laboratoire d’Ecologie et de Management des Ecosystèmes Aquatiques (LEMEA), Département de Zoologie, Faculté des Sciences et Techniques, Université d’Abomey-Calavi, Cotonou, Benin

**Keywords:** Conservation biology, Molecular ecology, Evolutionary biology, Population genetics

## Abstract

Inland fisheries are an important source of protein and income for people in Africa. Their sustainable management can greatly benefit from identification of regional genetic stocks and characterization of their genetic diversity, but such information is lacking for most African freshwater fisheries. The African bonytongue, *Heterotis niloticus*, is an important component of inland fisheries in West Africa. Nigeria has the largest fishery for African bonytongues, representing ~ 86% of the global total. Recent declines in yields at some Nigerian locations, however, suggest current levels of exploitation may be unsustainable. Habitat degradation also may be impacting some stocks. Despite its commercial and nutritional importance, the African bonytongue has been the subject of scant genetic research to support management. We examined patterns of genetic diversity in natural populations of *H. niloticus* at four locations in Nigeria, including Kainji Lake, a reservoir on the Niger River in north-central Nigeria, and three southern localities (Ethiope River, Igbokoda River, and Epe Lagoon), as well fish from the Ouémé River delta near Porto Novo, Benin. Eighty-five specimens were genotyped for nine microsatellite-loci. Genetic diversity estimates were highest at Kainji Lake, and substantially lower at southern localities. High levels of genetic differentiation were detected between samples from Kainji Lake and those from southern localities. Low, yet significant *F*_*ST*_ values were observed among samples from southern Nigerian localities that were more differentiated from the sample from nearby coastal Benin. We thus recommend that African bonytongues from the five locations be considered distinct genetic stocks and managed accordingly.

## Introduction

Inland fisheries provide an important source of protein, employment, and income for people in Africa^1–4^. Capture inland fisheries in Africa produced an estimated 3,004,437 metric tons in 2018, an amount only surpassed by that from Asia at 7,953,840 metric tons^5^. Inland fisheries in Africa, however, are facing growing threats that include overexploitation, habitat degradation, and introduction of exotic species that can impact native fish stocks^6,7^. Given that human populations and economies are growing rapidly in Africa, impacts from these threats are expected to increase^7,8^. The human population in sub-Saharan Africa is projected to double by 2050^9^, and it is estimated that one in every four people will be undernourished in this region^10^. Inland fisheries will continue to play a critical role in supporting food security and livelihoods in growing populations and economies of the continent. Therefore, it is important to conduct research that informs the sustainable use of these resources. Documentation of genetic diversity patterns of inland fisheries species is needed for identification of management and/or conservation units and for monitoring fishing impacts^11^. At present, genetic studies are very limited for most African freshwater fisheries, even though such studies have been long recognized as a research priority for the sustainable exploitation of these valuable resources^12^.

Nigeria is the most populous country in Africa, ranking 7th among nations globally (with an estimated population of ~ 206 million). In addition, Nigeria is one of nine countries that collectively will contribute more than half the projected growth of the global population between now and 2050^9^. Freshwater fish are important in the diet of Nigerians^2^, and demand for fish has increased considerably with population growth. An estimated 420,078 metric tons of freshwater fish were caught in Nigeria in 2017 and 392,188 metric tons in 2018, ranking first and second in harvest, respectively, among African countries^13^. These quantities are nearly four-fold greater than in 1980, when Nigeria’s population was ~ 73.4 M, and 107,530 metric tons of freshwater fishes were captured. Nigeria is one of the seven countries that are driving most of the growth in global inland fisheries^2^, and by 2050 could be one of the world's largest consumers of fish^14^. Abundance of many freshwater fishes in Nigeria, however, appears to be declining due to overfishing^15^. Of the 266 freshwater fish species documented in 2005, 47 (17%) were reported to be critically endangered, 15 (5%) endangered, 8 (3%) vulnerable, 23 (8%) near threatened, and the status of 129 (48%) was unknown^6^.

The African bonytongue, *Heterotis niloticus* (Cuvier, 1829), is a freshwater fish widely distributed in tropical rivers and freshwater lakes of Western and Central Africa, and the Nile Basin, where it supports important commercial and subsistence fisheries^16^. The African bonytongue has been introduced for aquaculture at several locations across Africa^17^, and is considered one of the top ten species for future use in global aquaculture^18^. The greatest harvest of African bonytongues occurs in Nigeria^13^. Between 1990 and 2019, ~ 362,534 metric tons of this fish were caught in Nigeria, corresponding to 6.6% of the national freshwater fish yield, and ~ 83.4% of the total yield for this species from the entire continent. Recent bonytongue yields represent a sharp increase in comparison to previous decades. For example, in 2010, 15,392 metric tons were caught in Nigeria, representing 85.2% of the total yield for this species from the continent, and 5.2% of the national freshwater fish yield; whereas in 2000, only 7915 metric tons were caught in Nigeria. Current levels of exploitation of this species may be unsustainable in regions of Nigeria where catches appear to be declining^19^.

The only prior research on the population genetics of the African bonytongue was conducted in Benin, the country on the western border of Nigeria^20^. That study detected high levels of genetic differentiation among fish sampled from three river basins: (1) the Niger River at Malanville, a city in northern Benin located upstream from where the river enters Nigeria; (2) a location on the lower Mono River in coastal south-west Benin; and (3) nine localities in the Ouémé-Sô floodplain system in south-central Benin. Within the Ouémé–So river-floodplain system, however, low levels of genetic differentiation were detected among the localities sampled, suggesting that seasonal flooding facilitates gene flow. Low genetic variability was detected for African bonytongues at Malanville, in the Niger River, compared to fish from the Mono and Ouémé-Sô basins^20^. Malanville is a fishing community where some stocks may be overexploited^21^. Therefore, it is important to examine other localities in the Niger Basin to determine whether low genetic diversity is a characteristic of *H*. *niloticus* in that system.

**Figure 1:**
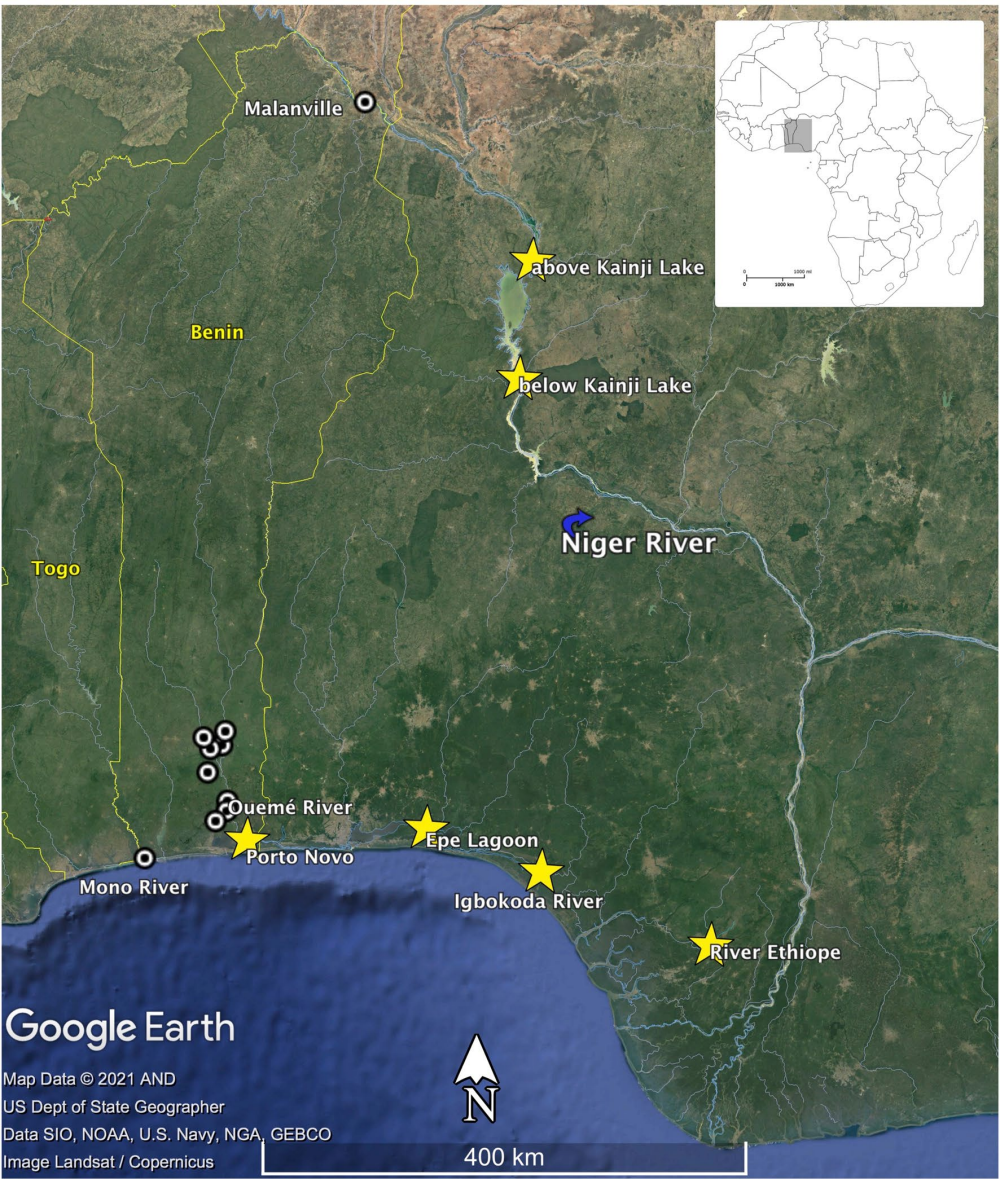
Map of the study area. Yellow stars indicate the collection sites of *Heterotis niloticus* for this study: Kainji Lake (10° 42′ N; 4° 42′ E and 9° 50′ N; 4° 37′ E); Ethiope River (5° 53′ N; 5° 43′ E), Igbokoda River (6° 17′ N; 4° 49′ E); Epe Lagoon (6° 34′ N; 3° 59′ E); Porto-Novo (6° 27′ N; 2° 37′ E). White circles show collection sites of *H. niloticus* in the study of Hurtado et al. (2013) in Benin.

Because of the significance of the African bonytongue fishery in Nigeria, and its potential for genetic differentiation, it is critical to evaluate genetic patterns within exploited stocks and to identify appropriate management units. Fishing can erode genetic diversity of inland fish stocks; thus, estimation of genetic diversity is important for assessment of population viability and evolutionary potential. Herein, we examined genetic diversity and differentiation among natural populations of *H. niloticus* from four locations in Nigeria where fishing activity is intense. These include Kainji Lake, in north central Nigeria, a reservoir on the Niger River formed by the Kainji Dam, and three localities in southwestern Nigeria close to the Atlantic coast: Ethiope River, Igbokoda River, and Epe Lagoon (Fig. 1). We also analyzed samples from the lower Ouémé River near Porto Novo in coastal southern Benin. Kainji Lake is approximately 230 km downstream from Malanville and approximately 700 km upstream from the massive Niger River delta, which forms ~ 240 km before the river empties into the Atlantic. Within the delta, the Niger River channel divides to form an intricate network of waterways surrounded by extensive floodplains that spread along the coast for ~ 320 km and cover an area of ~ 36,000 km^2^. The Ethiope and Igbokoda rivers are located in the western portion of the delta. Epe Lagoon, which is close to the Igbokoda River, and Porto Novo are located west of the delta. Given the large distance separating Kainji Lake and the southern localities, we hypothesized there would be very little gene flow, and therefore relatively large genetic differentiation between samples from the two areas. Furthermore, Kainji Dam, which was completed in 1968, provides an effective barrier to upstream fish movement, although the spillway may allow for some degree of downstream passage. Relatively low levels of genetic differentiation are expected among southern coastal localities where high fluvial connectivity and annual flooding could facilitate fish dispersal.

## Methods

### Sampling and DNA extractions

Samples of wild *H. niloticus* specimens were obtained from four inland waters in Nigeria: Kainji Lake (including samples from an area just above the lake and a location below the dam), Igbokoda River, Epe Lagoon and Ethiope River (Fig. 1). Additional specimens were obtained from the Ouémé River floodplain near Porto Novo, Benin. Dead fish were purchased from local commercial fishermen, and in some cases only pectoral fin clips were obtained from fish at the point of sale to consumers. Animal ethics approval was not required for this study because live fish samples were not collected. A pectoral fin clip from each specimen was stored in an Eppendorf tube with 70% ethanol. Genomic DNA was extracted from the pectoral fin clips of the tissue samples using the Qiagen DNeasy Blood & Tissue Kit (Valencia CA).

### Microsatellites amplification and genotyping

Samples were genotyped for nine microsatellite loci (Table SM1) reported for the African bonytongue^20,22^. Amplification of the extracted DNA was accomplished through multiplexing using Qiagen’s Multiplex PCR kit (Multiplex PCR Kit, QIAGEN, Valencia, CA) carried out in 10 µl reaction volumes containing 50–1000 ng genomic DNA, 5 µl QIAGEN Multiplex PCR Master Mix (containing HotStarTaq® DNA Polymerase, Multiplex PCR buffer and dNTP Mix), 1 µl of the forward (fluorescent labelled) and reverse primer mix and 3 µl of nuclease-free water (Qiagen Sample and Assay Technologies, Germany). Amplifications were carried out using a BIO-RAD Thermal Cycler (T100TM) programmed according to standard multiplex PCR protocols as follows: initial activation step: 15 min at 95 °C, 35 cycles of denaturation at 94 °C for 30 s, annealing at 57 °C for 90 s, extension at 72 °C for 60 s, and a final extension step 60 °C for 30 min. PCR products were run on 2% agarose gel electrophoresis to check for amplification of all loci prior to genotyping, and positive PCR products were then run on an ABI 3130xl automated sequencer using Genescan 400 HD ROX as size standard (Applied Biosystems). Allele calling and sizing for each sample was obtained using Genemarker software (version 1.75).

### Genetic diversity and population differentiation analyses

To convert input files between software packages, we used PGDSpider v. 2.1.1.3^23^. Tests of linkage disequilibrium (LD) and test of conformity to the expectations of Hardy–Weinberg Equilibrium proportions (HWP) were performed using GENEPOP on the Web 4.7^24,25^. To control for the occurrence of false positives due to multiple comparisons, we used the Benjamini–Hochberg procedure^26^ at a false discovery rate (FDR) of 0.05 for assessing the significance of LD and HWP *p* values. All GENEPOP analyses were performed with a dememorization number of 10,000 and 1000 batches with 10,000 iterations per batch. GenAIEx v. 6.5^27,28^ was used to calculate the following genetic diversity statistics: number of private alleles (*N*_*P*_), number of private alleles average number of alleles (*Na*), average number of effective alleles (*Ne*), Shannon’s information index (*I*), mean observed heterozygosity (*H*_*O*_), mean expected heterozygosity (*He*), unbiased expected heterozygosity (*uHe*), and inbreeding coefficient (*F*_*IS*_). We used MICRO-CHECKER v. 2.2.3^29^ to check for the potential presence of null alleles and scoring errors.

Population genetic structure was analyzed using several different approaches. Pairwise population *F*_*ST*_ values, with and without correction for null alleles, and their corresponding 95% confidence intervals (CI) were estimated using the program FreeNA^30^. *F*_*ST*_ values in which the 95% CIs excluded zero were deemed statistically significant. Pairwise population *F*_*ST*_ values were also estimated using the program Arlequin v.3.5.2.2^31^, which does not correct for null alleles, and their corresponding *p* values were estimated with 10,000 permutations. The Benjamini–Hochberg procedure at a false discovery rate (FDR) of 0.05 was used to determine whether these *F*_*ST*_ values were statistically significant. A discriminant analysis of principal component analysis (DAPC) with the R package *adegenet* v.2.1.1^32,33^ was conducted to visualize levels of genetic differentiation among the samples from the different locations, defining sampling locations as a priori groups. DAPC is a non-model-based method that maximizes the differences between groups while minimizing variation within groups^34^; and distances between genetic clusters using a priori groupings reflect underlying *F*_*ST*_^35^. STRUCTURE 2.2.3^36^, which performs model-based clustering with a Bayesian approach, was also used to examine population subdivision. Two models were used: admixture with correlated allele frequencies, and no admixture with correlated allele frequencies. *K* values from 1 to 5 were tested in ten iterations, with 500,000 steps and a burn-in of 125,000 steps, and all other settings were set to default. GenAlEx was used to construct a genetic distance matrix, from which a principal coordinates analysis (PCoA) was performed to identify population clusters.

Finally, we analyzed the relationship between genetic differentiation and geographic distance by testing for isolation by distance (IBD) using ISOLDE in GENEPOP on the Web 4.7. Two kinds of analyses were performed: (1) between localities; and, (2) between individuals. We used Google Earth Pro to measure distances between sample locations. For the distances between locations within the southern rivers, we measured the shortest straight-line distance. For distances between the Kainji Lake and all other localities, we followed the contour of the Niger River to the closest point to the Ethiope River, and from there we used straight lines. Statistical significance based on the Spearman’s rank correlation coefficient was evaluated using Mantel tests^37^.

## Results

### Genetic diversity

Genotyping scores for all individuals are shown in Dataset S1. Complete genotypes for the nine microsatellite-loci were obtained for 83 specimens: 23 from Kainji Lake, 19 from Ethiope River, 15 from Igbokoda, 20 from Epe Lagoon, and six from Porto Novo (Table 1). No linkage disequilibrium was detected among loci. A total of 98 individual alleles were observed across these 83 specimens; and the number of alleles per locus ranged from 4 in locus *Hn45* to 19 in *Hn30* (Table SM2). Departures of Hardy–Weinberg equilibrium were detected for locus *Hn47* in Kainji Lake, and locus *Hn11* in Igbokoda. According to MICROCHECKER, only locus *Hn11* in the Igbokoda sample shows signs of a null allele, which is suggested by a general excess of homozygotes for most allele size classes. Excess of homozygotes at this locus, however, may reflect inbreeding at this locality (see below).

**Table 1:** Genetic diversity estimates for *H. niloticus* from five localities in Nigeria and Benin.

Locality	*N*	*N*_*A*_	*N*_*P*_	*Na*	*Ne*	*I*	*Ho*	*He*	*uHe*	*F*
Kainji	23	75	21	8.33	4.56	1.60	0.73	0.70	0.72	− 0.03
Ethiope River	19	44	2	4.89	2.58	1.01	0.50	0.50	0.51	− 0.01
Igbokoda	15	51	4	5.67	2.55	1.11	0.47	0.54	0.56	0.18
Epe Lagoon	20	46	3	5.11	2.65	0.97	0.44	0.47	0.48	0.06
Porto Novo	6	32	3	3.56	2.49	0.93	0.57	0.49	0.53	− 0.16

*N*, number of individuals; *N*_*A*_, number of alleles; *N*_*P*_, number of private alleles; *Na*, average number of alleles per locus; *Ne*, average number of effective alleles per locus; *I*, Shannon’s information index; *Ho*, mean observed heterozygosity; *He*, mean expected heterozygosity; *uHe*, mean unbiased expected heterozygosity; *F*, inbreeding coefficient (fixation index).

The sample from Kainji Lake revealed the highest genetic diversity among the five populations examined (Table 1): average number of alleles (*Na*) = 8.33; average number of effective alleles (*Ne*) = 4.56; Shannon’s information index (*I*) = 1.6; observed heterozygosity (*Ho*) = 0.73; expected heterozygosity (*He*) = 0.70; and unbiased expected heterozygosity (*uHe*) = 0.72. The highest number of private alleles was also observed at Kainji Lake: 21 out of 75 alleles in this locality. This sample had a low inbreeding coefficient (*F*_*IS*_ = –0.03). Genetic diversity in the samples from the three southern Nigerian populations were substantially lower: *Na* ranged between 4.89 (Ethiope River) and 5.67 (Igbokoda); *Ne* between 2.55 (Igbokoda) and 2.65 (Epe Lagoon); *I* between 0.97 (Epe Lagoon) and 1.11 (Igbokoda); *Ho* between 0.44 (Epe Lagoon) and 0.50 (Ethiope River); *He* between 0.47 (Epe Lagoon) and 0.54 (Igbokoda); *uHe* between 0.48 (Epe Lagoon) and 0.56 (Igbokoda). In these localities, *F*_*IS*_ ranged between –0.01 (Ethiope River) and 0.18 (Igbokoda). Excluding locus *Hn11* in Igbokoda, for which null alleles were suggested in this locality, *F*_*IS*_ was 0.12, still the highest value. Thus, it is possible that the excess of homozygotes in *Hn11* observed in Igbokoda results from inbreeding rather than null alleles. Between two and four private alleles were observed in the southern Nigerian populations (out of 44, 51, and 46 alleles observed in Ethiope River, Igbokoda, and Epe Lagoon, respectively). The sample from the Ouémé River in Porto Novo, Benin, had the lowest allelic diversity (3.56, 2.49 and 0.93 for *Na*, *Ne* and *I*, respectively); although *Ho* (0.57) was higher than the southern Nigerian localities, and *He* (0.49) and *uHe* (0.53) were within the range observed in these localities. Three private alleles were observed at this locality (out of 32). The lowest inbreeding coefficient was estimated for the sample of this locality (*F*_*IS*_ = -0.16). Estimates of genetic diversity per locus per population is presented in Table SM2.

### Population genetic structure

*F*_*ST*_ values obtained with the program FreeNA were practically identical with and without correction for null alleles, indicating that null alleles, if present, did not bias results. The highest *F*_*ST*_ values were obtained for the pairwise comparisons that included Kainji Lake (Table 2). Average *F*_*ST*_ for these comparisons correcting for null alleles was 0.18. Average *F*_*ST*_ for pairwise comparisons between southern Nigerian localities correcting for null alleles was 0.05. Average *F*_*ST*_ for the pairwise comparisons between southern Nigeria localities and the Ouémé River in Benin was 0.11. All pairwise 95% CI excluded zero; thus, they are considered to be statistically significant. Similarly, all pairwise comparisons were significant according to the FDR test (FDR ≤ 0.05), using the *p* values obtained for the pairwise comparisons without correcting for null alleles.

**Table 2:** Pairwise *F*_ST_ estimations with (above the diagonal) and without correction for null alleles (95% confidence intervals in brackets; *p* values below brackets; * =  < 0.0001).

Population	Kainji Lake	Ethiope River	Igbokoda	Epe Lagoon	Porto Novo
Kainji Lake		0.17 [0.11–0.24]	0.15 [0.11–0.19]	0.21 [0.15–0.29]	0.19 [0.09–0.30]
Ethiope River	0.18 [0.11–0.24] *		0.03 [0.01–0.04]	0.08 [0.03–0.14]	0.09 [0.04–0.14]
Igbokoda	0.17 [0.12–0.21] *	0.02 [0.004–0.03] *		0.05 [0.02–0.08]	0.12 [0.06–0.17]
Epe Lagoon	0.22 [0.15–0.29] *	0.08 [0.03–0.14] *	0.04 [0.01–0.07] *		0.13 [0.06–0.23]
Porto Novo	0.19 [0.09–0.30] *	0.09 [0.04–0.14] 0.0001	0.12 [0.06–0.17] 0.0001	0.13 [0.06–0.23] *	

**Figure 2:**
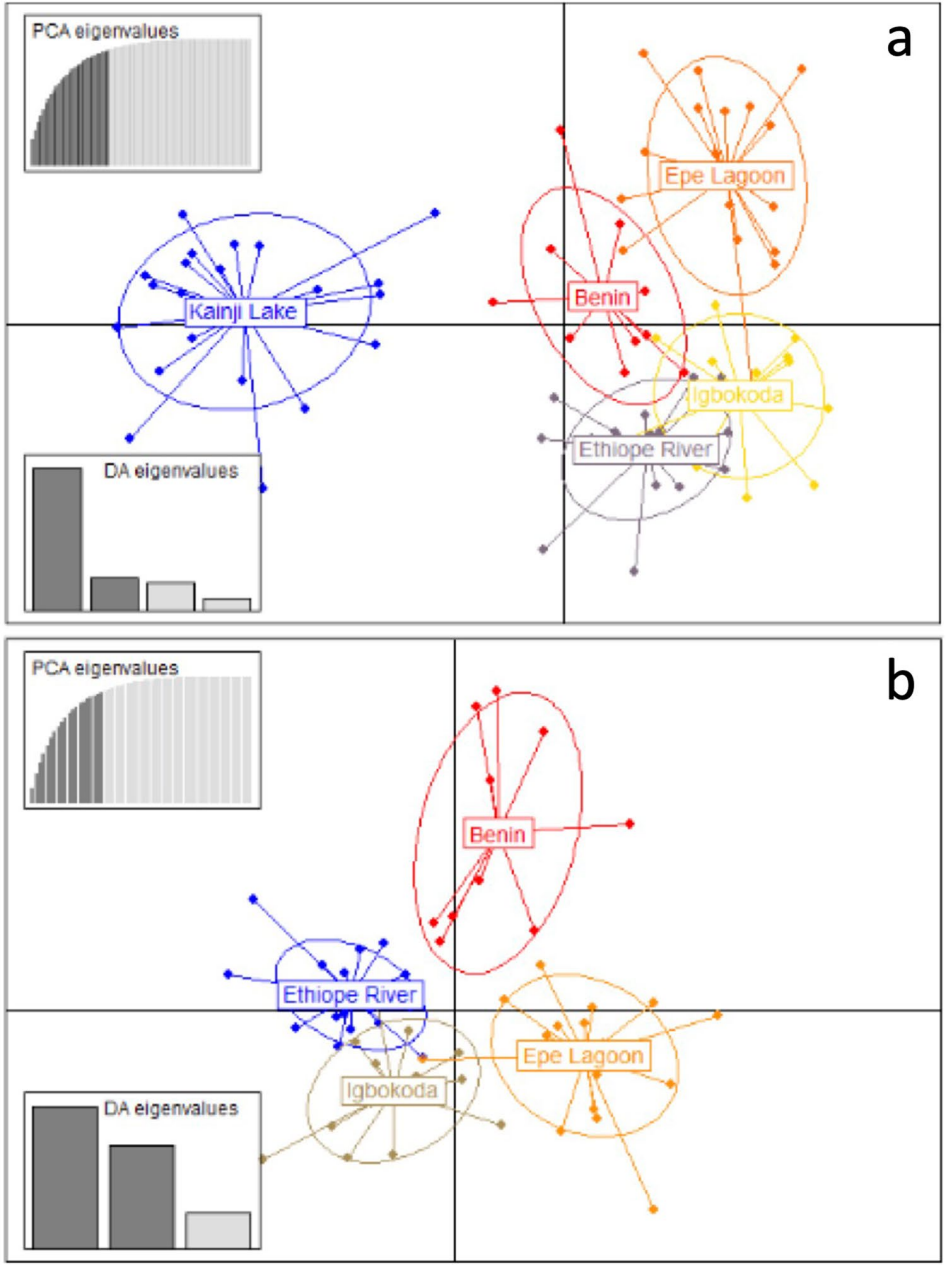
Cross-validated DAPC scatter plots (dots represent individuals) using sampling locations as a priori groups: (**a**) including all individuals from the five sampling locations (30 PCs); (**b**) including only the individuals from the southern populations (20 PCs).

**Figure 3:**
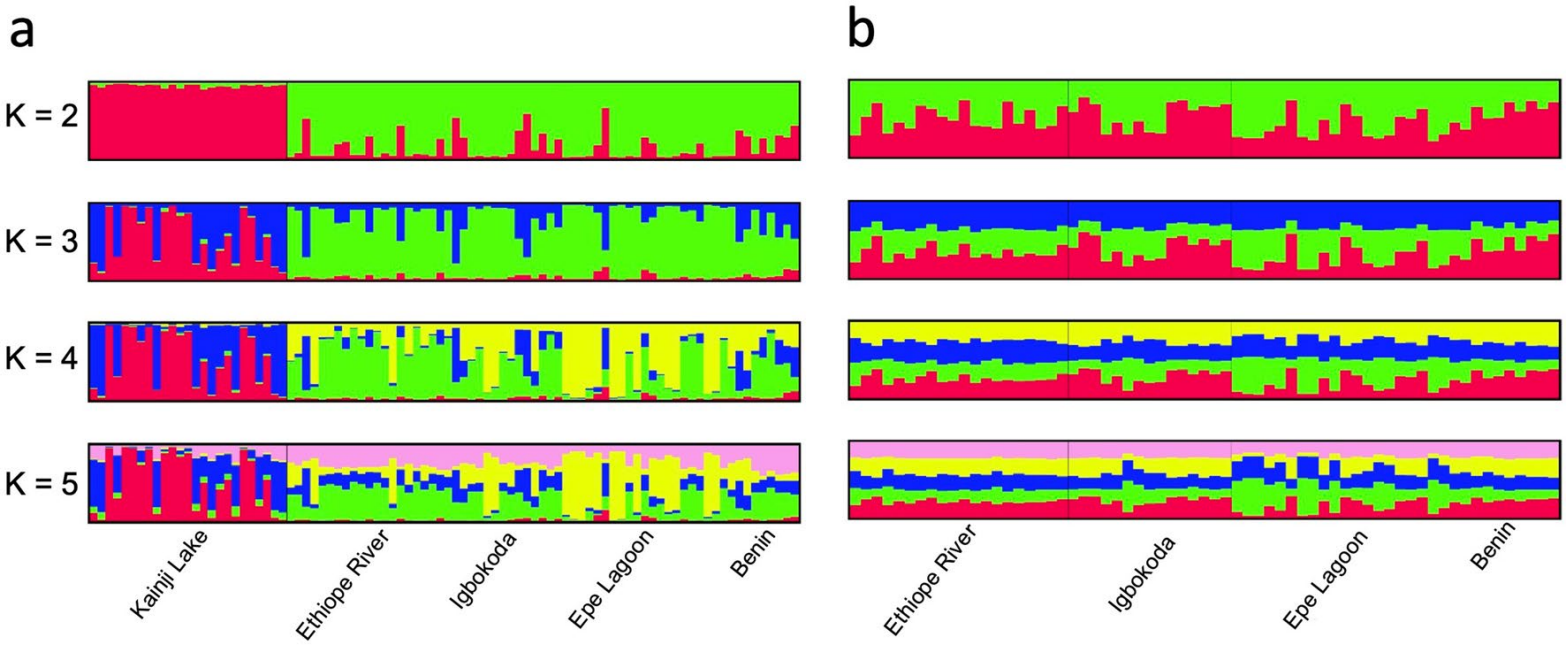
STRUCTURE bar plots of posterior probability for individual assignments using the admixture-correlated model for *K* = 2 to *K* = 5: (**a**) including all individuals from the five sampling locations; (**b**) excluding individuals from Kainji Lake.

**Figure 4:**
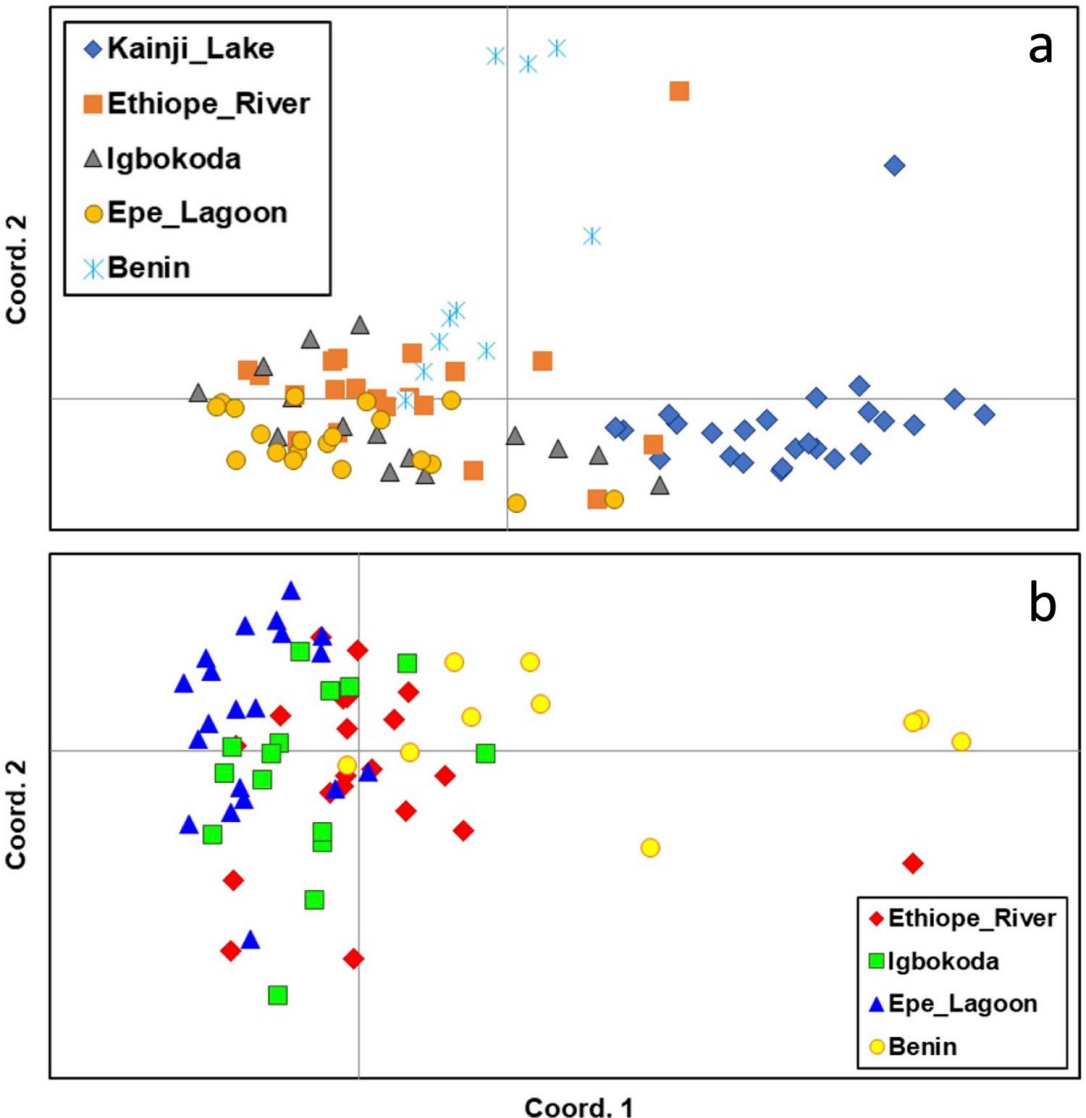
Principal coordinate analyses (PCoA) based on pairwise genetic distances of individual multilocus genotypes (individuals are color-coded by sampled population and plotted on the first two coordinates): (**a**) including all individuals from the five sampling locations; (**b**) excluding individuals from Kainji Lake.

**Figure 5:**
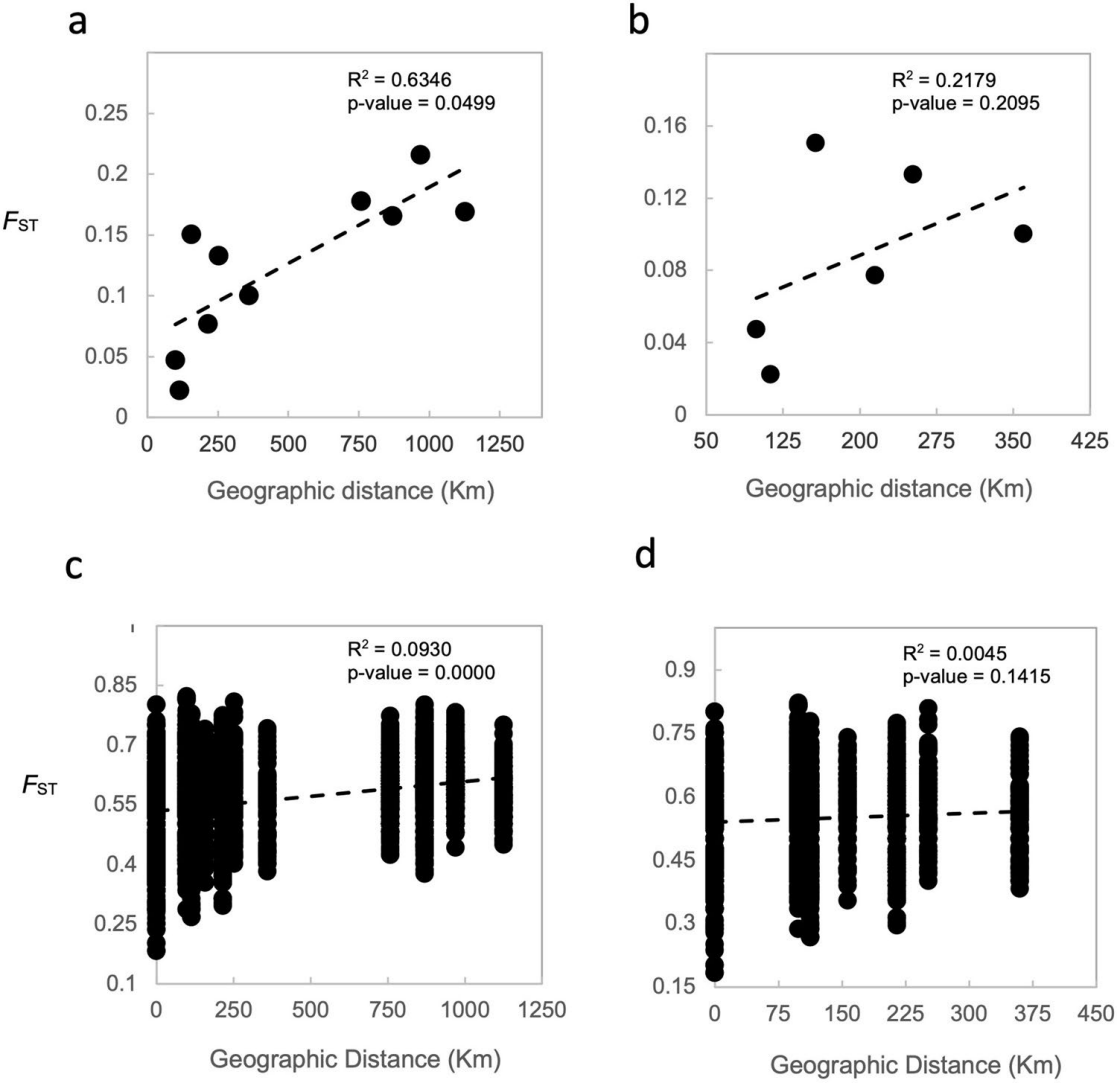
Isolation by distance (IBD) analyses. Correlation between geographic distance and genetic divergence *F*_ST_ for: (**a**) all five sampling locations; (**b**) the four southern sampling locations (i.e., excluding Kainji Lake); (**c**) considering all 83 individuals sampled separately; and (**d**) considering the 60 individuals from the southern sampling locations separately.

Distances between clusters in DAPC plots reflect *F*_*ST*_ results (Fig. 2), with the greatest distances observed between fish from Kainji Lake and fish from the southern localities. When the sample from Kainji Lake was removed, the greatest distances observed were between fish from the Ouémé River locality in Benin and fish from the three southern Nigeria localities. Fish from Epe Lagoon appear to be more genetically differentiated from conspecifics obtained from the other two southern Nigerian localities, and some overlap is observed between individuals from Igbokoda and Ethiope River. Analyses in STRUCTURE and PCA (Figs. 3 and 4) also show a clear separation between fish from Kainji Lake and fish from the other localities; however, they do not show a separation among fish samples from the different southern localities. The STRUCTURE plot in Fig. 3 includes two additional specimens from the Kainji Lake sample for which partial genotypes were obtained (one with four and one with five microsatellite-loci), and they grouped with all other specimens from Kainji Lake. The sample from Kainji Lake contained 3–4 individuals that were collected at Faku, just below the dam. For analyses excluding the Kainji Lake sample, STRUCTURE and PCA plots do not show evidence of genetic differentiation among fish from the southern localities. IBD analysis using *F*_*ST*_ values between populations produced an R^2^ = 0.65 with a *p* value = 0.049 for the analysis including all localities (Fig. 5a), whereas the analysis excluding Kainji Lake resulted in an R^2^ = 0.22 with a *p* value = 0.21 (Fig. 5b). IBD analyses using genetic differentiation between individuals resulted in an R^2^ = 0.09 with a *p* value < 0.0001 for the analysis including all localities (Fig. 5c), whereas the analysis excluding individuals from Kainji Lake resulted in an R^2^ = 0.005 with a *p* value = 0.21 (Fig. 5d).

## Discussion

Even though genetic evidence has long been recognized as crucially relevant for sustainable management of fisheries^11,38^, such information is lacking for most African inland fisheries^12^. The present study advances understanding of population genetics of the African bonytongue, an important fish for commerce and subsistence throughout a large region of Africa. We documented patterns of genetic diversity and structure of African bonytongue in Nigeria, where > 85% of the global capture of this fish occurs. The knowledge generated by the present study is important for guiding management and conservation strategies for this species.

The greatest genetic differentiation was observed between fish from Kainji Lake and those collected in the southern localities (average *F*_*ST*_ = 0.18). Although high, this level of differentiation is smaller than that observed (average *F*_*ST*_ = 0.27) between fish from Malanville, also in the Niger River, and southern Benin localities in Mono River and the Ouemé- Sô river floodplain system^20^. Differentiation between fish from Kainji Lake and those collected in the southern localities was supported by results from all genetic differentiation analyses, as well as the high number of private alleles (21) observed in Kainji Lake. The large geographic distance between Kainji Lake and southern populations is probably an important factor restricting gene flow of the African bonytongue between the two regions. A pattern of IBD was detected only when analyses included the sample of Kainji Lake. In addition, movements of the African bonytongue must be affected by the presence of the Kainji Dam, completed in 1968^39^. The dam constitutes an effective barrier for upstream movement of fishes from the river below, although fish can move downstream from the lake through the spillway. Nonetheless, the dam appears to have impacted downstream populations after its construction. Bonytongues were rarely caught at Faku, just below the dam, and Awuru, ~ 25 km downstream from the dam, in 1973 and 1974^39^. Our sample from Kainji Lake included a few specimens from Faku, and our genetic analyses grouped them in the same cluster as fish obtained from upstream of the lake, suggesting gene flow occurs from the lake to reaches just below the dam. The African bonytongue also became less frequent in landings in the second half of 1968 and in 1969 at Jebba and Pategi, ~ 100 km and ~ 220 km below the dam, respectively^40^. Between 1970 and 1972, however, bonytongues comprised a large percentage of the fish biomass landed at these locations, as well as at Lokoja, ~ 400 km downstream the dam. This proportion dropped substantially in these three below-dam localities between 1974 and 1975^39^, which may be due to local exploitation. It is important to investigate genetic differentiation of African bonytongues at these below-dam localities, where this species has historically been a valuable fishery component.

Comparisons between microsatellite results for bonytongues from Malanville in Benin^20^ and Kainji Lake (this study) suggest restricted gene flow between these two localities on the Niger River separated by a river distance of ~ 230 km. Microsatellite datasets for these samples were not analyzed together because of potential bias when combining datasets from different studies^41^. Marked differences in mean expected heterozygosity (*He*) for these samples, however, suggest highly restricted gene flow between these two areas of the Niger River. *He* was 0.57 in Malanville (n = 12), whereas *He* was 0.73 in the sample from Kainji Lake (n = 23). Mean *He* is expected to be fairly insensitive to potential bias due to differences in sample size^42,43^, and can be useful for comparisons between different studies^44^.

Among southern locations, gene flow appears to be more restricted between the lower Ouémé River in Benin (Porto Novo) and the southern Nigeria rivers sampled (average *F*_*ST*_ = 0.11). This level of differentiation is higher than the one reported in southern Benin between fish from Mono River and localities in the Ouemé- Sô river floodplain system (average *F*_*ST*_ = 0.09). Average divergence among southern Nigeria localities (average *F*_*ST*_ = 0.05) is higher than the reported between localities within the Ouemé- Sô river floodplain system (average *F*_*ST*_ = 0.03). A pattern of IBD was not detected among southern localities in this study. An ocean connection and the presence of brackish waters in Lagos Lagoon, which is connected to the western portion of Epe Lagoon, may represent effective barriers to gene flow for *H. niloticus* between Porto Novo and Epe Lagoon. According to our results, gene flow between upstream areas of the Ouémé River Basin and rivers connecting to Epe Lagoon also appears restricted. Significant pairwise *F*_*ST*_ values among the southern Nigerian localities suggest restricted gene flow among them, despite potential surface water connections provided by a complex network of waterways and annual flooding. Igbokoda River and Epe Lagoon are connected through a water channel that joins the Lekki Lagoon, which is connected to the eastern portion of Epe Lagoon. Thus, it appears that although movement of fish between the two localities may occur, it is not enough to prevent subtle genetic differentiation. Igbokoda and Ethiope rivers are separated by > 150 km. Flooding and water channels may facilitate movement of fish between both localities, although the lower portion of the Igbokoda River has brackish water that may act as a barrier to dispersal. STRUCTURE analyses did not detect subdivision among fish from southern locations, even though simulations have shown that this method can detect substructure in cases where *F*_*ST*_ values are as low as 0.03 when ten highly variable microsatellite loci are used^45^. Thus, it is possible that the microsatellite loci we used in this study are not variable enough for STRUCTURE to detect substructure with *F*_*ST*_ values of 0.11. In the previous study from Benin, most STRUCTURE analyses did not detect genetic differentiation between fish from the Mono River and those from localities in the Ouemé- Sô River floodplain system, even though all *F*_*ST*_ pairwise values were significant (average *F*_*ST*_ was 0.09), and other methods also clearly showed this differentiation^20^.

Studies of large-scale patterns of genetic structure of other important capture fisheries are limited in West Africa. A recent study of the Nile tilapia *Oreochromis niloticus* analyzed samples collected from 23 localities across eight West African countries, representing the major catchments of the Volta, Niger, Senegal and Gambia River basins^46^. That study found a pattern of IBD among all localities, and significant spatial genetic structure that largely corresponds to major river basins and, to a lesser extent, sub-basins. Within the Volta Basin, a significant, yet much weaker relationship between genetic and geographic distances was observed, suggesting IBD was a relatively minor factor shaping genetic differentiation amongst populations. This is similar to our findings for the African bonytongue, for which IBD was significant at a large scale (i.e., when Kainji Lake samples were included), but not at smaller scales (i.e., when only samples of southern populations were included). Most of the pairwise *F*_*ST*_ values in the Nile tilapia study were significant, except for pairwise comparisons among localities separated by some of the shortest distances. Significant genetic differentiation in the Nile tilapia was usually observed between populations separated by more than ~ 90 km. Interestingly, Nile tilapia samples from the only two localities sampled in the Niger River, Malanville and Mopti, which are separated by ~ 1400 km, show high genetic similarity, suggesting high levels of gene flow. This contrasts with what we observed for the African bonytongue in the Niger River, for which high genetic differentiation appears to occur at comparatively shorter distances within this river, i.e., between Malanville and Kainji Lake (~ 230 km), and between Kainji Lake and the lower Niger portion (~ 700 km).

An effect of floodplain connectivity and geographic scale has also been reported in the African bonytongue’s closest living relative, *Arapaima gigas*^47^. This species, which is distributed throughout the Amazon River Basin, is the only other living member of the Arapaiminae^48^, and both species construct nests where they lay and protect eggs. The predatory arapaima protects free-swimming larvae much longer (several weeks) than the omnivorous African bonytongue (a few days), and grows much larger (over 2 m and 100 kg compared to *Heterotis* at 1 m and 10 kg). At a fine scale (e.g. within the same floodplain system; < 25 km in *Arapaima* and ~ 75 km in *Heterotis*), the two species tend to exhibit genetic homogeneity^20,47^. At a meso-scale (e.g. in separate floodplain systems; ~ 100 km in *Arapaima* and 69–400 km in *Heterotis*), both species exhibit low but significant values of genetic differentiation. Finally, at the largest scale (e.g. > 1300 km in *Arapaima* and > 510 km in *Heterotis*), the highest levels of genetic differentiation were observed in both species. Thus, despite occupying two separate continents these two sister taxa exhibit similar patterns of genetic differentiation.

A broad range of genetic diversity values are observed in African bonytongue populations of Nigeria and Benin. *Na* values for this fish are similar or much lower than the average (*Na* = 9.1) reported for freshwater fishes^49^. Heterozygosity includes values considerably higher or lower than the average heterozygosity (*H* = 0.54) reported for freshwater fishes^49^. The sample from Kainji Lake had the highest heterozygosity (*Ho* = 0.73; *He* = 0.70) and second highest allelic diversity (*Na* = 8.33) among all African bonytongue populations examined to date. The sample from the Oueme–Sô river-floodplain system in Benin has the highest *Na* (9.25) for African bonytongues, but with a much larger sample examined (n = 184), and the second highest heterozygosity (*Ho* = 0.60; *He* = 0.69)^20^. Genetic diversity values for fish from the southern Nigeria localities are much lower (*Na* range = 4.89–5.67; *Ho* range = 0.44–0.50; *He* range = 0.47–0.54), and one of these localities, the Igbokoda River sample, shows high levels of inbreeding (*F*_*IS*_ = 0.18). The high genetic diversity in Kainji Lake contrasts with the low genetic diversity observed at Malanville, Benin (*Na* = 3.50; *Ho* = 0.34; *He* = 0.43), the only other Niger River locality sampled to date, and where high inbreeding was also observed (*F*_*IS*_ = 0.20)^20^. Low genetic diversity at Malanville could be associated with intense fishing pressure^20^. Although we observed low allelic diversity (*Na* = 3.56) for the sample from the Oueme–Sô River at Porto Novo, Benin, *Ho* (0.57) was more similar to the value previously reported for the Oueme–Sô river-floodplain, and *He* (0.49) was similar to that observed for samples from southern Nigeria. The smaller *Na* and *He* values obtained for Porto Novo compared to the Oueme–Sô river-floodplain system are likely due to the small (n = 6) sample size^43^. Values of *Na* more similar to the one obtained in Porto Novo were previously obtained for some localities with small sample sizes within the Oueme–Sô river floodplain system^20^. For a sample of n = 6 in the Ouemé River channel, values were *Na* = 3.38, *Ho* = 0.48 and *He* = 0.66; whereas for a sample of n = 10 in the Sô River channel, values were *Na* = 3.62, *Ho* = 0.55 and *He* = 0.61.

### Implications for conservation and management

The observed patterns of genetic differentiation indicate that African bonytongue from each of the localities examined in this study correspond to differentiated populations (i.e., genetic stocks), and should thus be treated separately for conservation and management. The Kainji Lake population is highly differentiated from the southern populations. In the south, the sample from Porto Novo is highly differentiated from southern Nigerian populations. Porto Novo is part of the Ouemé- Sô river floodplain system in Benin, for which Hurtado et al.^20^ report low levels of genetic differentiation among fish collected from this system, with sampling localities separated by up to ~ 75 km (average *F*_*ST*_ = 0.03). In that study, the fish from the Ouemé- Sô river floodplain system were highly differentiated from those collected in the Mono River and the Niger River locality of Malanville. The southern Nigerian populations sampled in this study show subtle, albeit statistically significant differentiation; we thus recommend that they be managed as local stocks. These genetically distinct populations could constitute valuable genetic resources for future use in aquaculture.

Multiple activities threaten the sustainability of the African bonytongue stocks we have identified in Nigeria. Overfishing compromises the sustainability of fishery resources in Kainji Lake^50,51^, and illegal fishing activities, including the use of prohibited gear (e.g. small mesh size nets and destructive fishing gear), fish poisoning, and explosives, have exacerbated overfishing in this lake^52^. Environmental pollution also impacts fish and people who consume them; and high levels of heavy metals have been detected in fish from the area^53^. The Ethiope River flows through a densely populated area of Nigeria’s Delta state, where pollution has impaired water quality in some stretches, with documented effects on macroinvertebrates^54^. Effects of degraded water quality in this river have not yet been shown to impair fish survival directly^55^. The Igbokoda River is an important fishing location in the Ilaje local government at Ondo State, a major oil-producing state. Unsafe levels of heavy metals have been found in water samples and fish in this river^56^. Consumption of fish is also considered unsafe in other rivers located in the Ilaje local government due to oil and industrial pollution^57^. Given the low quality of its riverine environment, and high levels of inbreeding detected in this study, urgent attention needs to be paid to the African bonytongue in Igbokoda River. A decade ago, Epe Lagoon was reported to have diverse and abundant fish stocks threatened by a rapidly growing population in the Lagos metropolitan area^58^. Anthropogenic activities appear to largely contribute to pollution in Epe Lagoon. Recent studies of Epe lagoon reported the presence of benzene, toluene, ethylbenzene and xylene (BTEX) and unsafe levels of Polycyclic Aromatic Hydrocarbons (PAH) in *H. niloticus*; as well as high levels of heavy metals in sediment of the lake, and a high prevalence of an intestinal parasite in *H. niloticus*, which may be attributable to the water pollution^59,60^.

## Conclusion and recomendations

Consistent with a previous study conducted in Benin, we found significant genetic structure among African bonytongue samples from locations in Nigeria. The highest values of genetic differentiation were observed between Kainji Lake and the southern localities examined. We found evidence of gene flow from Kainji Lake to the area just below the dam, but the large distance between Kainji Lake and southern populations probably restricts gene flow between the two regions. Relatively low but significant *F*_*ST*_ values were obtained for pairwise comparisons among fish from the southern localities. Based on these results, we suggest that populations of the African bonytongue in each of the Nigerian localities examined in this study be treated separately for conservation and management. Given the observed levels of genetic differentiation, understanding patterns of genetic diversity in the African bonytongue throughout the rest of its range is needed for adequate delineation of management stocks. We recommend that populations located in different rivers that are not part of the same floodplain system and are separated by > 70 km, or populations within a major river separated by > 200 km, be treated as separate management units pending genetic evidence.

## Supplementary Information

Supplementary Information 1.

Supplementary Table S1.

Supplementary Table S2.
