# Supplementary material for: Genetic identification and diversity of stocks of the African bonytongue, Heterotis niloticus (Osteoglossiformes: Arapaiminae), in Nigeria, West Africa
Source: Sci Rep. 2022 May 19;12:8417. doi: 10.1038/s41598-022-12428-6 (PMC9120501; doi:10.1038/s41598-022-12428-6)
Supplement: Supplementary file 2 — Supplementary Table S1. [file 41598_2022_12428_MOESM2_ESM.docx]

**Table SM1.** Primers sequences of the *H. niloticus* microsatellites loci used in this study

| **Microsatellite** | **Alternate name** | **Forward Primer Sequence (5^/^_-_ 3^/^)** | **Reverse Primer Sequence (5^/^_-_ 3^/^)** |
| --- | --- | --- | --- |
| **Hni5** | Hni5 | CAGGAAGATTTGCACCACCT**^FAM^** | TGCCATTCTGGAAAAAGGAG |
| **Hni28** | Hni28 | TCCCTGCAGTCTGAAACACA**^FAM^** | AGACCCACAAGATCCAGGTG |
| **Hn54** | Hni94 | GCCACCGTTTGAGTGAATTT**^HEX^** | TCTGAGAACACGGTAGGATGC |
| **Hn47** | Hni67 | CGCCTGTAAGCAATGAATGA**^FAM^** | TGCCATGGATGTCACAAGTT |
| **Hn32** | Hni19 | GGCTGTGCAATTTGACATG**^HEX^** | GTGCCTACAGCCATTTACCC |
| **Hn45** | Hni61 | AGCTTGCATCTGGAAGCATT**^FAM^** | GTATCCACCTGGACCCACAG |
| **Hn30** | Hni14 | CAACCACTGCCATAGGAGGT**^HEX^** | GAACCAAGTCGAGCCATCAT |
| **Hn14** | Hni52 | AAGGGGACCCTTGCAGTAGT**^HEX^** | GATGCAACATGCACTGCTCT |
| **Hn11** | Hni47 | AACACATTACGCCCTTGGAG**^FAM^** | CAGCCCTGTGTACACTTCCA |

*microsatellites names used in Carrera et al. (2011) and Hurtado et al. (2013)
