# Supplementary material for: Genetic identification and diversity of stocks of the African bonytongue, Heterotis niloticus (Osteoglossiformes: Arapaiminae), in Nigeria, West Africa
Source: Sci Rep. 2022 May 19;12:8417. doi: 10.1038/s41598-022-12428-6 (PMC9120501; doi:10.1038/s41598-022-12428-6)
Supplement: Supplementary file 3 — Supplementary Table S2. [file 41598_2022_12428_MOESM3_ESM.pdf]

Table SM2. Genetic diversity estimates for *H. niloticus* per locus per locality

Pop		Hn5	Hn11	Hn 14	Hn28	Hn 30	Hn32	Hn45	Hn47	Hn54
**Kainji_Lake**	**N**	23	23	23	23	23	23	23	23	23
	**Na**	9	14	11	8	14	5	3	6	5
	**Ne**	5.014	8.202	7.399	4.853	5.977	1.859	1.970	3.861	1.866
	**I**	1.795	2.348	2.159	1.739	2.184	0.965	0.753	1.503	0.977
	**Ho**	0.783	0.913	0.957	0.783	0.913	0.478	0.435	0.870	0.435
	**He**	0.801	0.878	0.865	0.794	0.833	0.462	0.492	0.741	0.464
	**uHe**	0.818	0.898	0.884	0.812	0.851	0.472	0.503	0.757	0.474
	**F**	0.022	-0.040	-0.106	0.014	-0.096	-0.035	0.117	-0.173	0.063
**Ethiope_River**	**N**	19	19	19	19	19	19	19	19	19
	**Na**	5	5	9	5	10	3	1	3	3
	**Ne**	2.498	1.656	5.309	2.983	4.688	1.715	1.000	1.383	1.994
	**I**	1.162	0.820	1.872	1.287	1.884	0.737	0.000	0.537	0.809
	**Ho**	0.632	0.421	0.789	0.737	0.789	0.526	0.000	0.211	0.421
	**He**	0.600	0.396	0.812	0.665	0.787	0.417	0.000	0.277	0.499
	**uHe**	0.616	0.407	0.834	0.683	0.808	0.428	0.000	0.284	0.512
	**F**	-0.053	-0.063	0.027	-0.108	-0.004	-0.262	#N/A	0.240	0.156
**Igbokoda**	**N**	15	15	15	15	15	15	15	15	15
	**Na**	6	7	8	7	9	5	2	4	3
	**Ne**	1.807	2.133	4.167	2.143	5.114	2.284	1.220	2.036	2.074
	**I**	0.996	1.184	1.629	1.202	1.864	1.066	0.325	0.951	0.802
	**Ho**	0.400	0.267	0.667	0.467	0.800	0.667	0.067	0.400	0.467
	**He**	0.447	0.531	0.760	0.533	0.804	0.562	0.180	0.509	0.518
	**uHe**	0.462	0.549	0.786	0.552	0.832	0.582	0.186	0.526	0.536
	**F**	0.104	0.498	0.123	0.125	0.006	-0.186	0.630	0.214	0.099
**Epe_Lagoon**	**N**	20	20	20	20	20	20	20	20	20
	**Na**	4	6	7	4	11	4	1	5	4
	**Ne**	1.229	2.424	4.571	1.444	6.452	3.042	1.000	1.299	2.424
	**I**	0.429	1.157	1.691	0.639	2.080	1.171	0.000	0.543	1.004
	**Ho**	0.200	0.500	0.650	0.250	0.850	0.600	0.000	0.200	0.700
	**He**	0.186	0.588	0.781	0.308	0.845	0.671	0.000	0.230	0.588
	**uHe**	0.191	0.603	0.801	0.315	0.867	0.688	0.000	0.236	0.603
	**F**	-0.074	0.149	0.168	0.187	-0.006	0.106	#N/A	0.130	-0.191
**Benin**	**N**	6	6	6	6	6	6	6	6	6
	**Na**	5	4	6	3	5	3	2	1	3
	**Ne**	3.273	2.880	5.143	2.667	2.571	2.323	1.180	1.000	1.412
	**I**	1.352	1.199	1.705	1.040	1.234	0.960	0.287	0.000	0.566
	**Ho**	0.833	0.833	1.000	0.833	0.500	0.667	0.167	0.000	0.333
	**He**	0.694	0.653	0.806	0.625	0.611	0.569	0.153	0.000	0.292
	**uHe**	0.758	0.712	0.879	0.682	0.667	0.621	0.167	0.000	0.318
	**F**	-0.200	-0.277	-0.241	-0.333	0.182	-0.171	-0.091	#N/A	-0.143
**Total all populations**	**Na**	14	14	17	10	19	6	4	8	6
